# Supplementary material for: Functional specialization of the subdomains of a bactofilin driving stalk morphogenesis in Asticcacaulis biprosthecum
Source: bioRxiv. 2024 Dec 16:2024.12.16.628611. Preprint. [Version 1] doi: 10.1101/2024.12.16.628611 (PMC11702518; doi:10.1101/2024.12.16.628611)
Supplement: Supplement 1 [file NIHPP2024.12.16.628611v1-supplement-1.pdf]

bioRxiv preprint doi: https://doi.org/10.1101/2024.12.16.628611; this version posted December 16, 2024. The copyright holder for this preprint (which was not certified by peer review) is the author/funder, who has granted bioRxiv a license to display the preprint in perpetuity. It is made available under aCC-BY 4.0 International license.

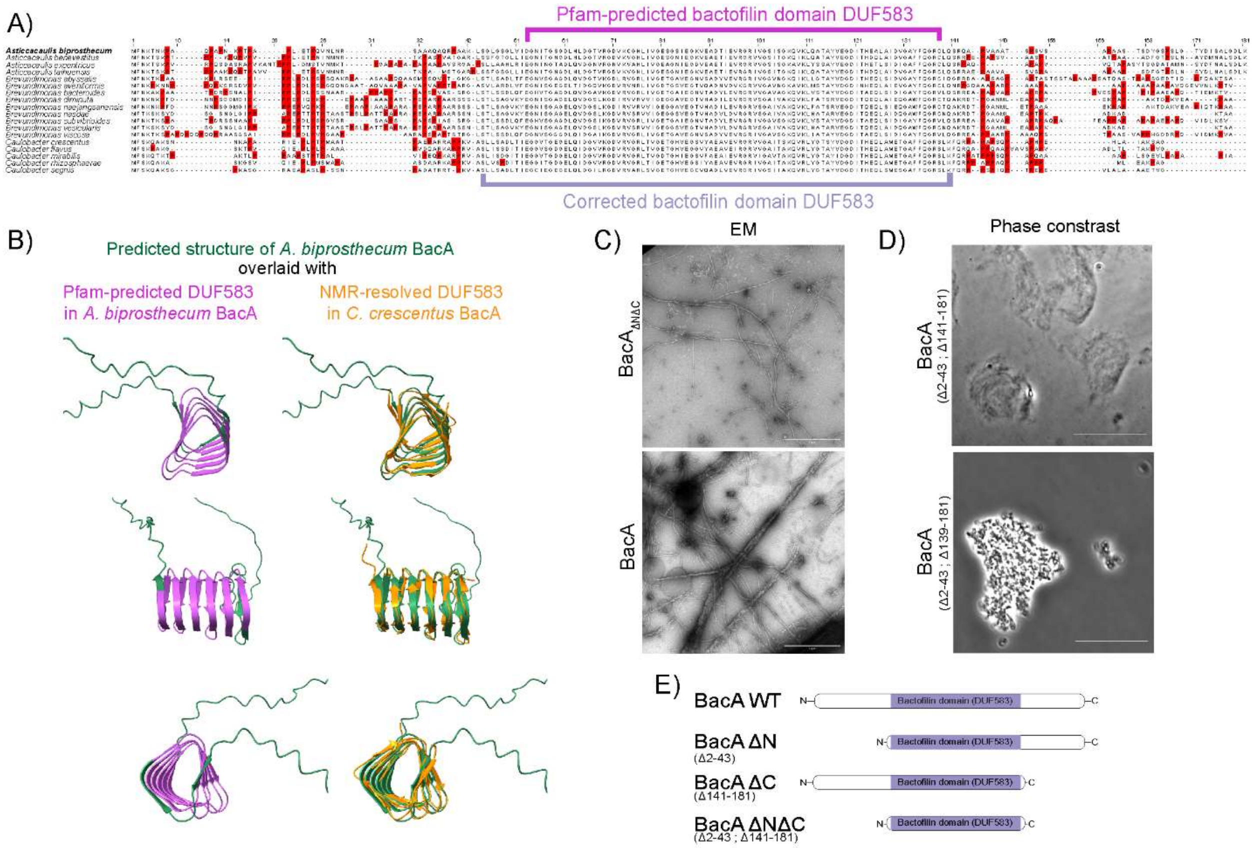

Figure S1 – Analysis of the domain structure of bactofilins in the *Caulobacteraceae* family, and proposed *A. biprosthecum* BacA mutations.

A) Multiple sequence alignment from Figure 1A highlighting proline residues (highlighted in red) in the proline-rich N- and C-terminal domains flanking the central bactofilin domain (DUF583). The Pfam-predicted bactofilin domain is delineated in magenta and the corrected bactofilin domain in mauve.

B) Left: The predicted structure of *A. biprosthecum* BacA superimposed with the NMR-resolved structure of *C. crescentus* BacA (orange) (PDB-ID : 2N3D). Right: Structural prediction of *A. biprosthecum* BacA (green) using AlphaFold, with the Pfam-delimited bactofilin domain (DUF583) in magenta. Side, top, and bottom views of the structures are presented.

C) High-resolution EM images of purified BacA and BacA$_{ΔNΔC}$ filaments (scale bar = 1 µm).

bioRxiv preprint doi: https://doi.org/10.1101/2024.12.16.628611; this version posted December 16, 2024. The copyright holder for this preprint (which was not certified by peer review) is the author/funder, who has granted bioRxiv a license to display the preprint in perpetuity. It is made available under aCC-BY 4.0 International license.

D) Phase contrast images of purified BacA$_{\Delta 2\text{-}43;\Delta 141\text{-}181}$ (BacA$_{\Delta N \Delta C}$) filaments and BacA$_{\Delta 2\text{-}43;\Delta 139\text{-}181}$ aggregates (scale bar = 20 µm).

E) Schematic of the full-length BacA protein from *A. biprosthecum* and the proposed mutants for this study, based on the corrected bactofilin domain (residues 44-140).

bioRxiv preprint doi: https://doi.org/10.1101/2024.12.16.628611; this version posted December 16, 2024. The copyright holder for this preprint (which was not certified by peer review) is the author/funder, who has granted bioRxiv a license to display the preprint in perpetuity. It is made available under aCC-BY 4.0 International license.

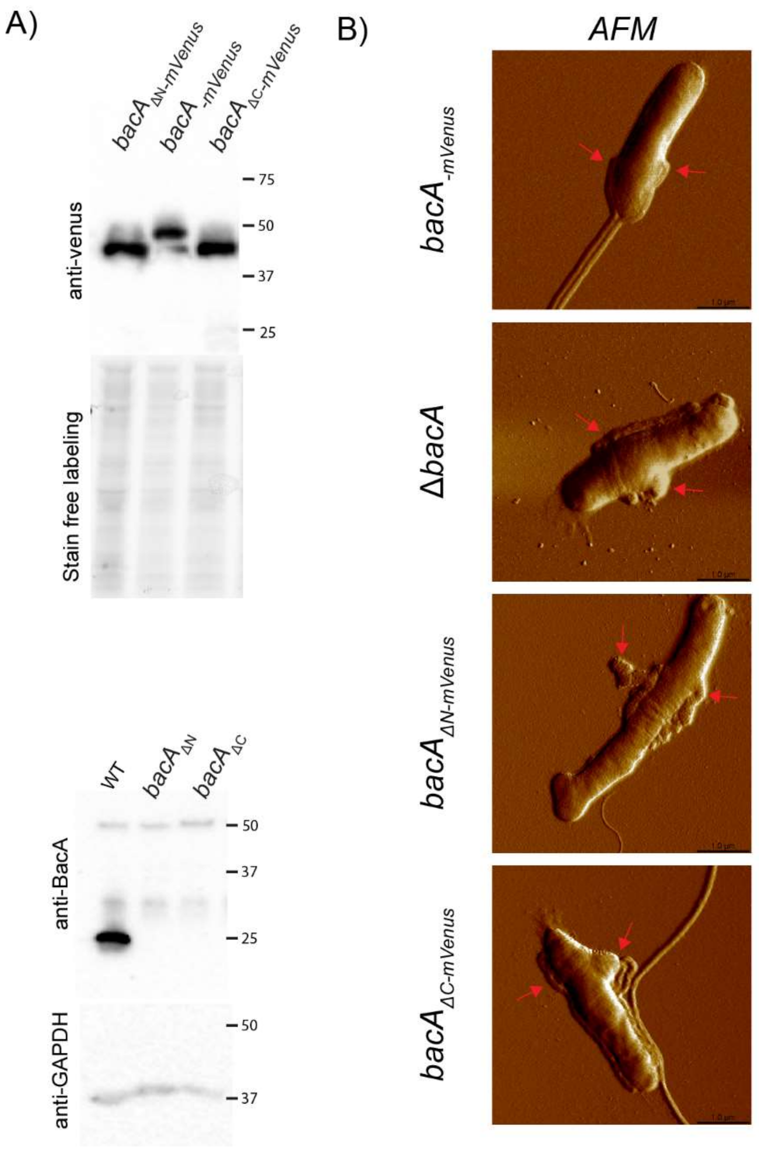

Figure S2 – Protein expression and AFM microscopy of BacA mutants strains

A) Top: Western blots using anti-GFP antibodies in mVenus-tagged BacA terminal domain deletion strains in *A. biprosthecum*. Strain free labeling was used as a loading control. Bottom: Western blots using anti-BacA antibodies in untagged terminal domain deletion strains in *A. biprosthecum* showed that antibodies raised against full length BacA protein were unable to detect truncated BacA mutants. Anti-GAPDH antibodies were used as a loading control.

bioRxiv preprint doi: https://doi.org/10.1101/2024.12.16.628611; this version posted December 16, 2024. The copyright holder for this preprint (which was not certified by peer review) is the author/funder, who has granted bioRxiv a license to display the preprint in perpetuity. It is made available under aCC-BY 4.0 International license.

B) Atomic force microscopy of *A. biprosthecum bacA-mVenus*, Δ*bacA*, *bacA*$_{\Delta N}$*-mVenus* and *bacA*$_{\Delta C}$*-mVenus* strains, used to analyze the width of the stalk base. Stalks/pseudostalks are indicated with red arrows. Cells were grown in phosphate-limited (HIGG) medium (see Methods). Scale bars = 1 μm.

bioRxiv preprint doi: https://doi.org/10.1101/2024.12.16.628611; this version posted December 16, 2024. The copyright holder for this preprint (which was not certified by peer review) is the author/funder, who has granted bioRxiv a license to display the preprint in perpetuity. It is made available under aCC-BY 4.0 International license.

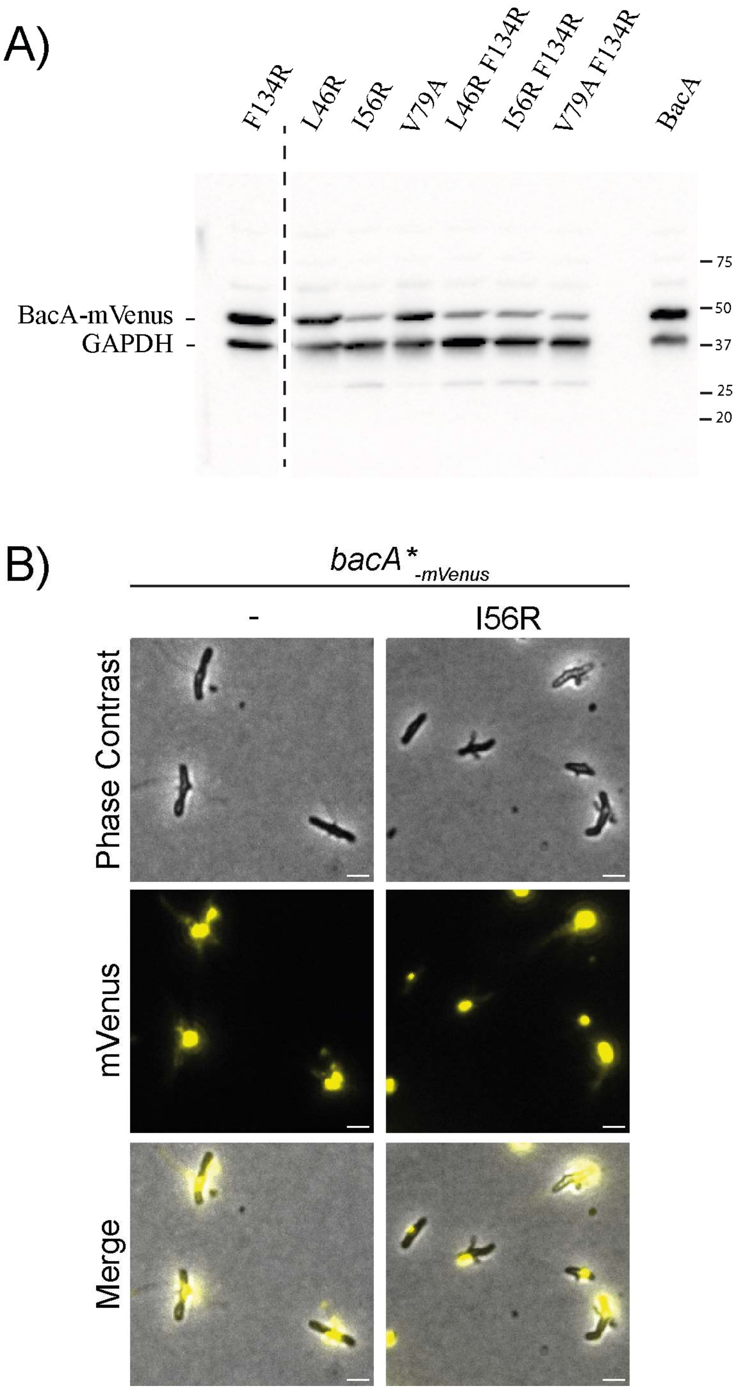

Figure S3 – Western blot to verify the expression of the BacA polymerization point mutants.

bioRxiv preprint doi: https://doi.org/10.1101/2024.12.16.628611; this version posted December 16, 2024. The copyright holder for this preprint (which was not certified by peer review) is the author/funder, who has granted bioRxiv a license to display the preprint in perpetuity. It is made available under aCC-BY 4.0 International license.

A) Western blot using anti-GFP antibodies in the BacA polymerization point mutants in *A. biprosthecum*. Cells lysates were loaded at the same level of protein. Anti-GAPDH is presented as a loading control.

B) Phase-contrast, fluorescence, and merged microscopy images of BacA-mVenus and BacA I56R-mVenus, with LUTs matching those used for mutants displaying diffuse fluorescence presented in Figure 5A.

bioRxiv preprint doi: https://doi.org/10.1101/2024.12.16.628611; this version posted December 16, 2024. The copyright holder for this preprint (which was not certified by peer review) is the author/funder, who has granted bioRxiv a license to display the preprint in perpetuity. It is made available under aCC-BY 4.0 International license.

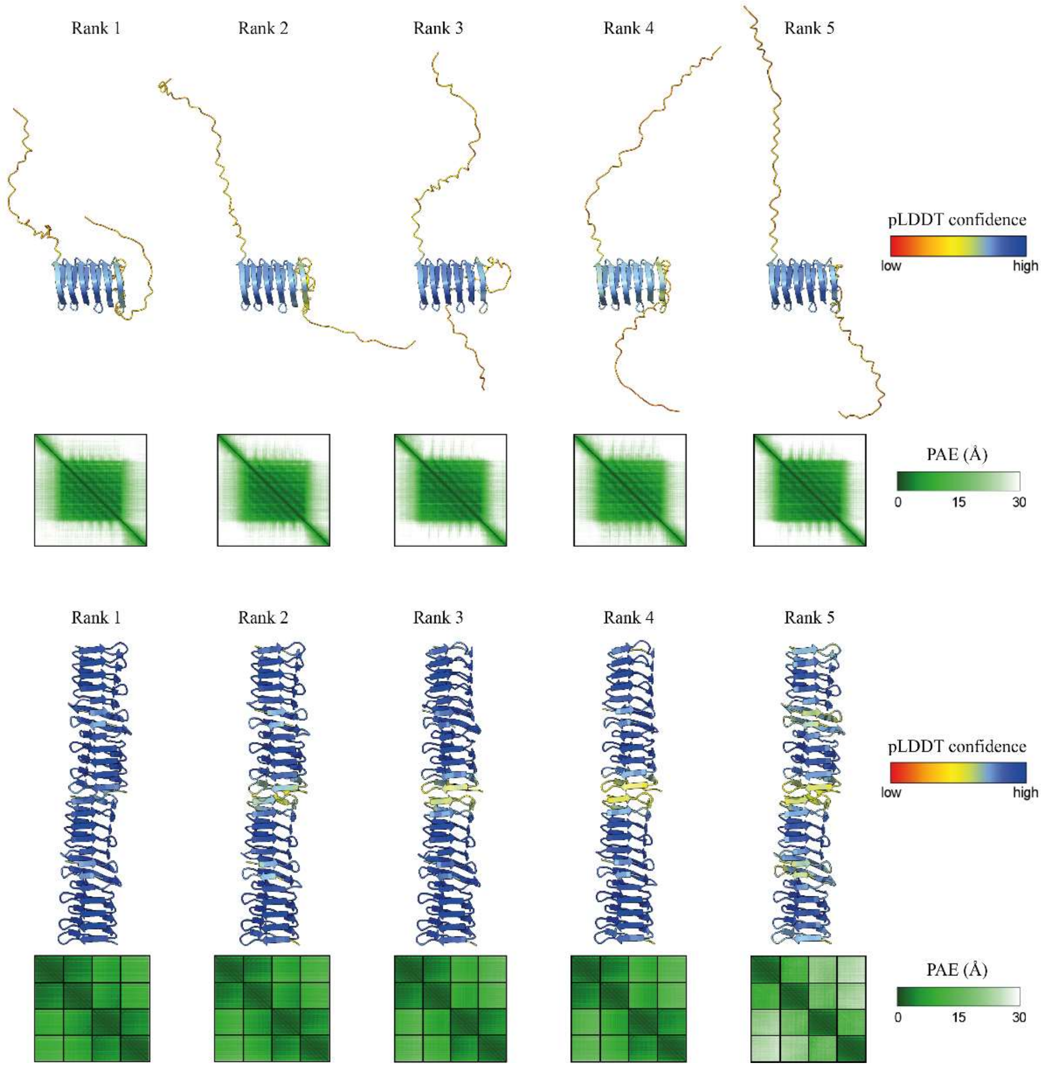

Figure S4: BacA monomer and tetramer structural predictions.

BacA monomer (top) and tetramer (bottom) predicted by AlphaFold, used in Figure 1 and 4 respectively. Structures are aligned and shown from the same orientation. Structures are ranked according to the predicted template modeling (pTM) score and are colored according to the predicted local distance difference test (pLDDT) score. Confidence in the prediction of each complex is indicated by the predicted aligned error (PAE) scores, which indicate positional error in angstroms for a given pair of residues across both protein chains.

Table S1 – Strains used in this study.

Strain (YB#)	Genotype/description	Construction	Reference/source
E. coli			
**105**	Overexpression strain; BL21λDE3 (F- ompT hsdSB (rB-mB-) gal dcm (IDE3)		-
**7351**	DAP auxotroph; WM3064 (thrB1004 pro thi rpsL hsdS lacZΔM15 RP4-1360 Δ(araBAD)567 ΔdapA1341::[erm pir])		(37)
**9171**	BACTH strain; BTH101 (F-, cya-99, araD139, galE15, galK16, rpsL1 (Str r), hsdR2, mcrA1, mcrB1.)		Euromedex EUB001
A. biprosthecum			
**642**	WT C19		(26)
**8597**	Δ*bacA*	-	(29)
**9139**	*bacA (aa 45-181)-mVenus (ΔN)*	Created by electroporating and double recombination using pMJ77 into A. biprosthecum C19.	This study
**9141**	*bacA-mVenus*	Created by electroporating and double recombination using pMJ79 into A. biprosthecum C19.	This study
**9189**	*bacA(aa 1-140)-mVenus (ΔC)*	Created by electroporating and double recombination using pMJ76 into A. biprosthecum C19.	This study
**9506**	*bacA F134R-mVenus*	Created by electroporating and double recombination using pMJ125 into A. biprosthecum C19.	This study
**9507**	*bacA L46R-mVenus*	Created by electroporating and double recombination using pMJ124 into A. biprosthecum C19.	This study
**9510**	*bacA I56R-mVenus*	Created by electroporating and double recombination using pMJ123 into A. biprosthecum C19.	This study
**9512**	*bacA V79A mVenus*	Created by electroporating and double recombination using pMJ159 into A. biprosthecum C19.	This study
**9513**	*bacA V79A F134R-mVenus*	Created by electroporating and double recombination using pMJ156 into A. biprosthecum C19.	This study
**9594**	*bacA L46A F134R-mVenus*	Created by electroporating and double recombination using pMJ127 into A. biprosthecum C19.	This study
**9595**	*bacA I56A F134R-mVenus*	Created by electroporating and double recombination using pMJ128 into A. biprosthecum C19.	This study

**9895**	bacA (aa 1-140) (ΔC)	Created by electroporating and double recombination using pMJ169 into A. biprosthecum C19.	This study
**9896**	bacA (aa 45-181) (ΔN)	Created by electroporating and double recombination using pMJ170 into A. biprosthecum C19.	This study
**10188**	bacA (aa 1-140) (ΔC); spmX-mCherry	Created by electroporating and double recombination using pNPTS138-SpmX-mCherry into A. biprosthecum YB9895.	This study
**10189**	bacA (aa 45-181) (ΔN); spmX-mCherry	Created by electroporating and double recombination using pNPTS138-SpmX-mCherry into A. biprosthecum YB9896.	This study
**10190**	spmX-mCherry	Created by electroporating and double recombination using pNPTS138-SpmX-mCherry into A. biprosthecum C19.	This study

Table S2 – Plasmids used in this study

Plasmid	*E.coli* YB#	Genotype/description	Reference/source
**Overexpression plasmids**			
pET28a+		Vector carrying an N-terminal His•Tag/thrombin site for overexpression in *E.coli*	Novagen Cat#69864
pMJ4	8539	pET28a+ derivative containing His-tagged BacA (ABI_34180) in *E. coli*; Kan^R	(29)
pMJ26	9052	pET28a+ derivative containing His-tagged F134R in *E. coli*; Kan^R	This study
pMJ66	9107	pET28a+ derivative containing His-tagged BacA aa45-138 in *E. coli*; Kan^R	This study
pMJ71	9108	pET28a+ derivative containing His-tagged BacA aa45-140 in *E. coli*; Kan^R	This study
pMJ109	9140	pET28a+ derivative containing His-tagged I56R in *E. coli*; Kan^R	This study
pMJ110	9138	pET28a+ derivative containing His-tagged I56R F134R in *E. coli*; Kan^R	This study
pMJ119	9306	pET28a+ derivative containing His-tagged L46R in *E. coli*; Kan^R	This study
pMJ120	9311	pET28a+ derivative containing His-tagged L46R F134R in *E. coli*; Kan^R	This study
pMJ132	9520	pET28a+ derivative containing His-tagged BacA aa9-181 in *E. coli*; Kan^R	This study
pMJ133	9522	pET28a+ derivative containing His-tagged V79A in *E. coli*; Kan^R	This study
pMJ157	9574	pET28a+ derivative containing His-tagged V79A F134R in *E. coli*; Kan^R	This study
**Bacterial two-hybrid plasmids**			
pKT25		Empty plasmid with MCS for C-terminal fusion with T25 fragment; Kan^R	Euromedex
pKNT25		Empty plasmid with MCS for N-terminal fusion with T25 fragment; Kan^R	Euromedex
pUT18		Empty plasmid with MCS for N-terminal fusion with T18 fragment; Amp^R	Euromedex
pUT18C		Empty plasmid with MCS for C-terminal fusion with T18 fragment; Amp^R	Euromedex
pKT25-zip		positive control with leucine zipper domain fused to T25 fragment; Kan^R	Euromedex
pUT-zip		Positive control with leucine zipper domain fused to T18 fragment; Amp^R	Euromedex
pPC62	9158	pKT25 derivative in which SpmX is fused to the T25 fragment; Kan^R	(29)
pPC63	9159	pKT25 derivative in which BacA is fused to the T25 fragment; Kan^R	(29)

pPC65	9162	pKNT25 derivative in which BacA is fused to the T25 fragment; Kan^R	(29)
pPC68	9165	pUT18 derivative in which BacA is fused to the T18 fragment; Amp^R	(29)
pPC72	9161	pKNT25 derivative in which SpmX is fused to the T25 fragment; Kan^R	(29)
pMJ105	9299	pKNT25 derivative in which BacA I56R is fused to the T25 fragment; Kan^R	This study
pMJ106	9300	pKNT25 derivative in which BacA I56R F134R is fused to the T25 fragment; Kan^R	This study
pMJ107	9301	pKT25 derivative in which BacA I56R is fused to the T25 fragment; Kan^R	This study
pMJ108	9302	pKT25 derivative in which BacA I56R F134R is fused to the T25 fragment; Kan^R	This study
pMJ111	9290	pUTC18 derivative in which BacA L46R F134R is fused to the T18 fragment; Amp^R	This study
pMJ114	9289	pUTC18 derivative in which BacA I56R is fused to the T18 fragment; Amp^R	This study
pMJ115	9307	pUTC18 derivative in which BacA I56R F134R is fused to the T18 fragment; Amp^R	This study
pMJ116	9288	pUT18 derivative in which BacA I56R is fused to the T18 fragment; Amp^R	This study
pMJ117	9314	pUTC18 derivative in which BacA I56R F134R is fused to the T18 fragment; Amp^R	This study
pMJ118	9315	pUTC18 derivative in which BacA L46R is fused to the T18 fragment; Amp^R	This study
pMJ136	9525	pKT25 derivative in which BacA ΔNΔC is fused to the T25 fragment; Kan^R	This study
pMJ137	9526	pKT25 derivative in which BacA ΔC is fused to the T25 fragment; Kan^R	This study
pMJ138	9527	pKNT25 derivative in which BacA ΔN is fused to the T25 fragment; Kan^R	This study
pMJ139	9528	pKT25 derivative in which BacA L46R is fused to the T25 fragment; Kan^R	This study
pMJ140	9529	pKT25 derivative in which BacA L46R F134R is fused to the T25 fragment; Kan^R	This study
pMJ142	9530	pKNT25 derivative in which BacA L46R is fused to the T25 fragment; Kan^R	This study
pMJ143	9559	pKNT25 derivative in which BacA L46R F134R is fused to the T25 fragment; Kan^R	This study
pMJ146	9560	pUT18 derivative in which BacA L46R is fused to the T18 fragment; Amp^R	This study
pMJ150	9561	pUT18 derivative in which BacA ΔN is fused to the T18 fragment; Amp^R	This study
pMJ151	9562	pUT18 derivative in which BacA ΔC is fused to the T18 fragment; Amp^R	This study
pMJ152	9563	pUT18 derivative in which BacA ΔNΔC is fused to the T18 fragment; Amp^R	This study
pMJ153	9564	pUT18C derivative in which BacA ΔN is fused to the T18 fragment; Amp^R	This study
pMJ154	9567	pUT18C derivative in which BacA ΔC is fused to the T18 fragment; Amp^R	This study
pMJ155	9568	pUT18C derivative in which BacA ΔNΔC is fused to the T18 fragment; Amp^R	This study
pMJ158	9569	pUT18C derivative in which BacA V79A is fused to the T18 fragment; Amp^R	This study

pMJ160	9570	pKNT25 derivative in which BacA ΔC is fused to the T25 fragment; Kan^R	This study
pMJ161	9571	pKT25 derivative in which BacA V79A is fused to the T25 fragment; Kan^R V79A	This study
pMJ162	9572	pUT18 derivative in which BacA V79A F134R is fused to the T18 fragment; Amp^R	This study
pMJ163	9575	pKNT25 derivative in which BacA V79A is fused to the T25 fragment; Kan^R	This study
pMJ164	9577	pKNT25 derivative in which BacA V79A F134R is fused to the T25 fragment; Kan^R	This study
pMJ165	9578	pUT18 derivative in which BacA V79A is fused to the T18 fragment; Amp^R	This study
pMJ166	9579	pKT25 derivative in which BacA ΔC is fused to the T25 fragment; Kan^R	This study
pMJ167	9580	pKNT25 derivative in which BacA V79A F134R is fused to the T25 fragment; Kan^R	This study
pMJ168	9581	pUT18C derivative in which BacA V79A F134R is fused to the T18 fragment; Amp^R	This study

Integration or expression plasmid in *A. biprosthecum*

pMJ75	9124	pNPTS138 derivative used to generate bacA (aa 45-140)-mVenus (ΔN ΔC); Kan^R	This study
pMJ76	9125	pNPTS138 derivative used to generate bacA (aa 1-140)-mVenus (ΔC); Kan^R	This study
pMJ77	9126	pNPTS138 derivative used to generate bacA (aa 45-181)-mVenus (ΔN); Kan^R	This study
pMJ78	9127	pNPTS138 derivative used to generate bacA-mVenus (ATG differed); Kan^R	This study
pMJ79	9128	pNPTS138 derivative used to generate bacA-mVenus; Kan^R	This study
pMJ123	9500	pNPTS138 derivative used to generate bacA I56R -mVenus; Kan^R	This study
pMJ124	9501	pNPTS138 derivative used to generate bacA L46R -mVenus; Kan^R	This study
pMJ125	9502	pNPTS138 derivative used to generate bacA F134R -mVenus; Kan^R	This study
pMJ127	9504	pNPTS138 derivative used to generate bacA L46R F134R -mVenus; Kan^R	This study
pMJ128	9505	pNPTS138 derivative used to generate bacA I56R F134R -mVenus; Kan^R	This study
pMJ131	9519	pNPTS138 derivative used to generate bacA (aa 9-181)-mVenus; Kan^R	This study
pMJ156	9573	pNPTS138 derivative used to generate bacA V79A F134R -mVenus; Kan^R	This study
pMJ159	9576	pNPTS138 derivative used to generate bacA V79A -mVenus; Kan^R	This study
pMJ169	9586	pNPTS138 derivative used to generate bacA (aa 1-140)(ΔC); Kan^R	This study
pMJ170	9587	pNPTS138 derivative used to generate bacA (aa 45-181) (ΔN); Kan^R	This study
pPC36	8584	pMR10 derivate containing the promotor region upstream of the ABI_34190/ABI_34180 loci fused to the coding region of ABI_34180; Kan^R	This study

pNPTS138-SpmX-mCherry	pNPTS138 derivative used to generate SpmX-mCherry; Kan^R	Vaidehi Patel
pNPTS138 1621	pLitmus derivative carrying *oriT* and *sacB*	M.R.K. Alley
